# Systematic evaluation of PAXgene® tissue fixation for the histopathological and molecular study of lung cancer

**DOI:** 10.1002/cjp2.145

**Published:** 2019-11-11

**Authors:** Mark Southwood, Tomasz Krenz, Natasha Cant, Manisha Maurya, Jana Gazdova, Perry Maxwell, Claire McGready, Ellen Moseley, Susan Hughes, Peter Stewart, Manuel Salto‐Tellez, Daniel Groelz, Doris Rassl, On behalf of the STRATfix Consortium

**Affiliations:** ^1^ Pathology Research Royal Papworth Hospital NHS Foundation Trust, University of Cambridge Clinical School of Medicine Cambridge UK; ^2^ Sample Technologies Department QIAGEN GmbH Hilden Germany; ^3^ Sample Technologies Department QIAGEN Ltd. Manchester UK; ^4^ Northern Ireland Molecular Pathology Laboratory Centre for Cancer Research and Cell Biology, Queen's University Belfast Belfast UK

**Keywords:** Histopathology, Lung Cancer, Immunohistochemistry, DNA sequencing

## Abstract

Whilst adequate for most existing pathological tests, formalin is generally considered a poor DNA preservative and use of alternative fixatives may prove advantageous for molecular testing of tumour material; an increasingly common approach to identify targetable driver mutations in lung cancer patients. We collected paired PAXgene® tissue‐fixed and formalin‐fixed samples of block‐sized tumour and lung parenchyma, Temno‐needle core tumour biopsies and fine needle tumour aspirates (FNAs) from non‐small cell lung cancer resection specimens. Traditionally processed formalin fixed paraffin wax embedded (FFPE) samples were compared to paired PAXgene® tissue fixed paraffin‐embedded (PFPE) samples. We evaluated suitability for common laboratory tests (H&E staining and immunohistochemistry) and performance for downstream molecular investigations relevant to lung cancer, including RT‐PCR and next generation DNA sequencing (NGS). Adequate and comparable H&E staining was seen in all sample types and nuclear staining was preferable in PAXgene® fixed Temno tumour biopsies and tumour FNA samples. Immunohistochemical staining was broadly comparable. PFPE samples enabled greater yields of less‐fragmented DNA than FFPE comparators. PFPE samples were also superior for PCR and NGS performance, both in terms of quality control metrics and for variant calling. Critically we identified a greater number of genetic variants in the epidermal growth factor receptor gene when using PFPE samples and the Ingenuity® Variant Analysis pipeline. In summary, PFPE samples are adequate for histopathological diagnosis and suitable for the majority of existing laboratory tests. PAXgene® fixation is superior for DNA and RNA integrity, particularly in low‐yield samples and facilitates improved NGS performance, including the detection of actionable lung cancer mutations for precision medicine in lung cancer samples.

## Introduction

Lung cancer is a leading cause of cancer mortality in the UK 1. Overall median survival is approximately 17 months, with fewer than 25% of cases suitable for surgical resection 2, 3. Moreover, most patients present with metastatic disease in which median survival falls to 6–9 months 4, 5. Multiple treatment strategies are employed, including ‘personalised’ or ‘stratified’ approaches, particularly for patients with non‐small cell lung cancer (NSCLC), whereby identification of driver genetic mutations to guide efficacious treatment is beneficial (for recent reviews see 6, 7, 8). To date, efforts have largely focussed upon targeting consequences of mutations in the epidermal growth factor receptor (*EGFR*) gene 9, 10, 11, 12, 13, 14, as well as *ALK* and *ROS1* translocations 15, 16, 17, although other genes, including *MET* gene amplification, may be of future benefit 18.

Technological innovations, including next generation DNA sequencing (NGS), to target‐sequence panels of causal cancer‐genes, now represent a practical modality for developed healthcare systems, including the UK National Health Service (NHS) 19, 20; however, current fixation protocols are often a bottle‐neck for molecular analysis and over/under‐fixation of samples is highly detrimental 21, 22, 23, 24. As many samples from lung cancer patients are physically small, low‐ and/or poor quality‐DNA yields can be hindered by fixation artefacts 25. Furthermore, health risks are associated with occupational exposure to formaldehyde 26, 27, 28.

These data are the findings of an Innovate UK‐funded study delivered by a partnership of NHS laboratories, academic institutions together with an industrial partner working to align advances in pre‐analytical processing with established workflows for handling pathological samples. Here we compared paired PAXgene® and formalin‐fixed samples from resection specimens and evaluated technical performance for histology, immunohistochemistry, DNA/RNA preservation and performance in molecular testing pertinent to the study of lung cancer.

## Methods

### Histopathological tumour sampling, fixation and processing schedules

This study was approved by the research ethics committee via the Royal Papworth Hospital Research Tissue Bank (08/H0304/56+5 and 18/EE/0269). Unfixed lung resection specimens from informed and consenting patients were sampled and paired block‐sized pieces of; (1) lung tumour and (2) background lung parenchyma were placed into buffered neutral formalin (Genta Medical, NewYork, UK) or PAXgene®‐tissue fixative (QIAGEN®, Manchester, UK). Similarly, paired PAXgene® and formalin fixed 16G Temno needle core biopsies (Careusion, BD, Wokingham, UK) of tumour material were collected from neighbouring tumour regions and fixed for 24–72 h.

Endobronchial or endoscopic ultrasound guided FNA samples are now accepted to be the preferred technique for the diagnosis and staging of advanced stage lung tumours 29, 30, 31, 32. To technically replicate these samples we aspirated tumour material from unfixed specimens using a wide‐bore needle and syringe, placed into either ThinPrep Cytolyt fixative (Hologic Inc., Manchester, UK) or PAXgene‐FNA® fixative (QIAGEN®, Manchester, UK) for >1 h. The plasma/thrombin clot preparation method is detailed in supplementary material, Supplementary materials and methods. Following fixation, all PAXgene® samples were transferred to PAXgene® stabiliser solution (QIAGEN®, Manchester, UK) and stored at −20 °C for batched formalin‐free processing. For complete processing schedules see supplementary material, Tables S1 and S2.

### H&E staining, histomorphological assessment and scoring

All samples were embedded in paraffin wax (60 °C) and blocks stored at −20 °C. Immediately prior to sectioning, blocks were removed from the freezer and 4 μm sections cut, stained with H&E and coverslipped (Multistainer, Leica, Milton Keynes, UK). Using a published scoring system, nuclear, cytoplasmic and cell membrane features were each assigned a score of 0–4 by two blinded observers (see 33 for details).

### Antigen retrieval and immunohistochemistry

Antigen retrieval is widely performed to counteract the effects of cross‐linking caused by formalin fixation. Despite PAXgene® being a non‐cross‐linking fixative, we opted to carry out antigen retrieval for both PFPE and FFPE material. Our reasoning for this was two‐fold; (1) to ensure a fair comparison between FFPE and PFPE samples and (2) laboratories opting to use PAXgene® technology might wish to harmonise FFPE and PFPE IHC protocols where possible. Antigen retrieval (20 min/96 °C) was performed with high pH antigen retrieval solution (PT module, DakoCytomation, Ely, UK) following the manufacturer's protocol. Immunohistochemistry was performed using batches of freshly prepared monoclonal anti‐human ‐Ki67, ‐MNF116, ‐p63, ‐cytokeratin 7 (CK‐7), ‐CK5/6, and ‐thyroid transcription factor‐1 (TTF‐1) antibodies. Negative controls consisted of slides undergoing pre‐treatment and incubation with antibody diluent followed by detection system reagents. Immunostaining was performed using the EnVision FLEX, high‐pH detection system and Autostainer Link48 Immunostainer, visualised using 3,3′‐diaminobenzidine tetrahydrochloride and counterstained using haematoxylin (all DakoCytomation, Ely, UK). Two independent observers scored the immunostaining, marking the sections out of 5 for intensity/specificity of staining; where 1 or 2 = unacceptable, 3 = borderline acceptable and 4 or 5 = acceptable. For the assessment of histomorphology, paired PFPE and FFPE pieces of block‐sized tumour samples, Temno biopsies and tumour FNA preparations were scored from a total of *n* = 23 patients and immunohistochemistry performed on all samples from *n* = 10 patients.

### Molecular testing of samples

Paired PFPE and FFPE samples from *n* = 8 subjects were referred to QIAGEN Ltd., Manchester, UK; QIAGEN GmbH, Hilden, Germany, and paired PFPE and FFPE samples from *n* = 23 patients (including the *n* = 8 also sent to QIAGEN) sent to Queens University Belfast (QUB). The samples comprised *n* = 10, 5 μm tissue sections on slides (for DNA) and *n* = 5, 10 μm tissue sections/curls (for RNA) in sterile 1.5 ml Eppendorf tubes (Eppendorf, Stevenage, UK) and shipped using standard UK Royal Mail 48 h delivery.

### QIAGEN analysis of DNA and RNA quality and integrity

Nucleic acids from all paired samples were extracted from FFPE samples using the QIAamp® DNA FFPE Kit and RNeasy® FFPE Kit (QIAGEN®, Manchester, UK) and from PFPE samples using the PAXgene® Tissue DNA or RNA kits (PreAnalytiX® GmbH, Hombrechtikon, Switzerland) following the manufacturers' protocols. DNA yield and purity (*A*_260 nm_/*A*_280 nm_) was measured using the NanoDrop 2000 Spectrophotometer and by QUBIT dsDNA Broad Range Fluorimetry (ThermoFisher Scientific, Hemel Hempstead, UK). DNA fragmentation was evaluated by Tapestation 2200 (Agilent, Stockport, UK).

### QIAGEN β‐actin RT‐PCR and *EGFR therascreen*® assays

Five microliter from the extracted RNA eluate was used in a 294 bp β‐actin real‐time RT‐PCR assay with reactions performed in duplicates. For primer sequences, see supplementary material, Supplementary materials and methods. DNA mutation analysis was performed using the *therascreen*® *EGFR* RGQ PCR Kit (QIAGEN, Manchester, UK) for detection of common exon 19–21 *EGFR* mutations using RT‐PCR, according to the manufacturer's instructions.

### QIAGEN Gene Reader™ NGS workflow including unique molecular indexing

DNA extracts from paired PFPE and FFPE samples were sequenced with the QIAGEN GeneReader™ NGS workflow. The GeneReader™ QIAact Lung DNA Panel (QIAGEN, Manchester, UK) was used for target enrichment PCR and library preparation from 40 ng of DNA following the manufacturer's protocol. The GeneReader™ workflow was used to compare quality control metrics for NGS performance. Unique molecular indexing (UMI) technology allows ligation of each original DNA molecule with a QIAact adapter containing a 12‐random base UMI prior to PCR amplification. Although developed to identify consequences of PCR or sequencing errors in NGS and to distinguish low frequency genetic variants from artefactual data it can also serve as a proxy measurement to compare how DNA preservation can affect sequencing coverage and fidelity between paired PFPE and FFPE samples.

### QUB analysis of DNA and RNA quality and integrity

Nucleic acids were extracted from FFPE specimens using the Maxwell 16 DNA/RNA Purification kit (Promega, Southampton, UK) and from PFPE specimens using the PAXgene® Tissue DNA/RNA kit (QIAGEN, Manchester, UK). Nucleic acid yield/purity was measured using the Qubit 2.0 Fluorometer and the Nanodrop 2000 Spectrophotometer (ThermoFisher, Hemel Hempstead, UK). Fragmentation of extracted DNA was measured using the Fragment Analyser Automated system for High Sensitivity Genomic DNA (DNF‐488 high sensitivity Genomic DNA kit) from Advanced Analytical (Ankeny, USA). 40–250 ng of extracted DNA was prepared for enrichment and library construction for the QIAseq Targeted DNA Panel assay protocol (QIAGEN, Manchester, UK). Libraries were prepared using the KAPA Biosystems quantification kit (Sigma‐Aldrich/Merck KGaA, Darmstadt, Germany) and NGS performed on Illumina MiSeq or Illumina NextSeq 500 sequencing platforms (Illumina, San Diego, CA, USA). This panel allows for sequencing of 72 genes commonly mutated in human lung cancer samples (see supplementary material, Table S3).

### Data summary and bioinformatics

Paired Student's *t*‐tests were used for comparisons between two groups. Multiple comparisons were assessed by one‐way ANOVA, followed by the appropriate *post‐hoc* test for significance. Data were analysed using the QIAGEN Targeted Sequencing Data Analysis Portal/GeneGlobe® Data Analysis Centre. Variants were called and annotated using the QIAGEN Ingenuity Variant Analysis software, and filtered for the following calling criteria: call quality of at least 30, read depth of at least 50 and a mutant allele fraction of at least 10%. FASTQ files were downloaded from BaseSpace and uploaded to the QIAGEN Data Analysis Centre and variants were called using QIAGEN Ingenuity® Variant Analysis software. The QIAGEN Clinical Insights Analyze (QCI®‐A) tool was applied for analysis of NGS results including QC parameters and variant calls. The DNA sequencing and bioinformatic pipeline is summarised in supplementary material, Figure S1.

## Results

### H&E and immunohistochemical staining of PFPE and FFPE samples

**Figure 1:**
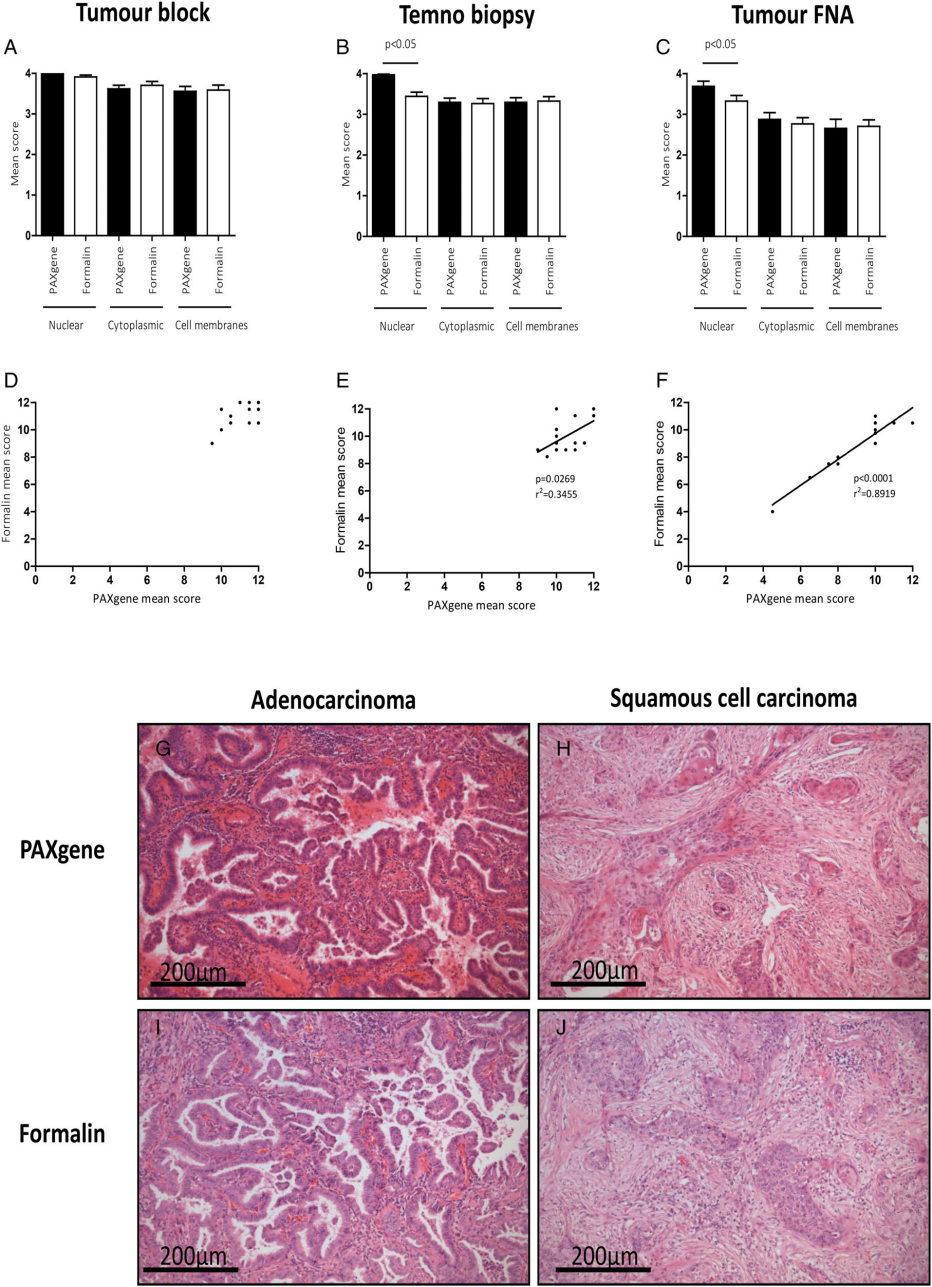
H&E staining and histomorphology. Histomorphology of PFPE and FFPE H&E stained sections of tumour block (A), Temno biopsy (B) and tumour FNA clot preparations (C). Nuclear staining of Temno biopsies and tumour FNA specimens was preferable in PFPE samples (both *p* < 0.05). Scores for nuclear, cytoplasmic and cell membranous staining were combined for tumour blocks (D), Temno biopsies (E) and for tumour FNA samples (F) and correlations between FFPE and PFPE scores were observed for Temno biopsies (*r* ^2^ = 0.3455, *p* = 0.0269) and tumour FNA samples (*r* ^2^ = 0.8919, *p* < 0.0001). Representative H&E staining of PFPE adenocarcinoma (G) and PFPE squamous cell carcinoma (H) and FFPE adenocarcinoma (I) and FFPE squamous cell carcinoma (J).

**Figure 2:**
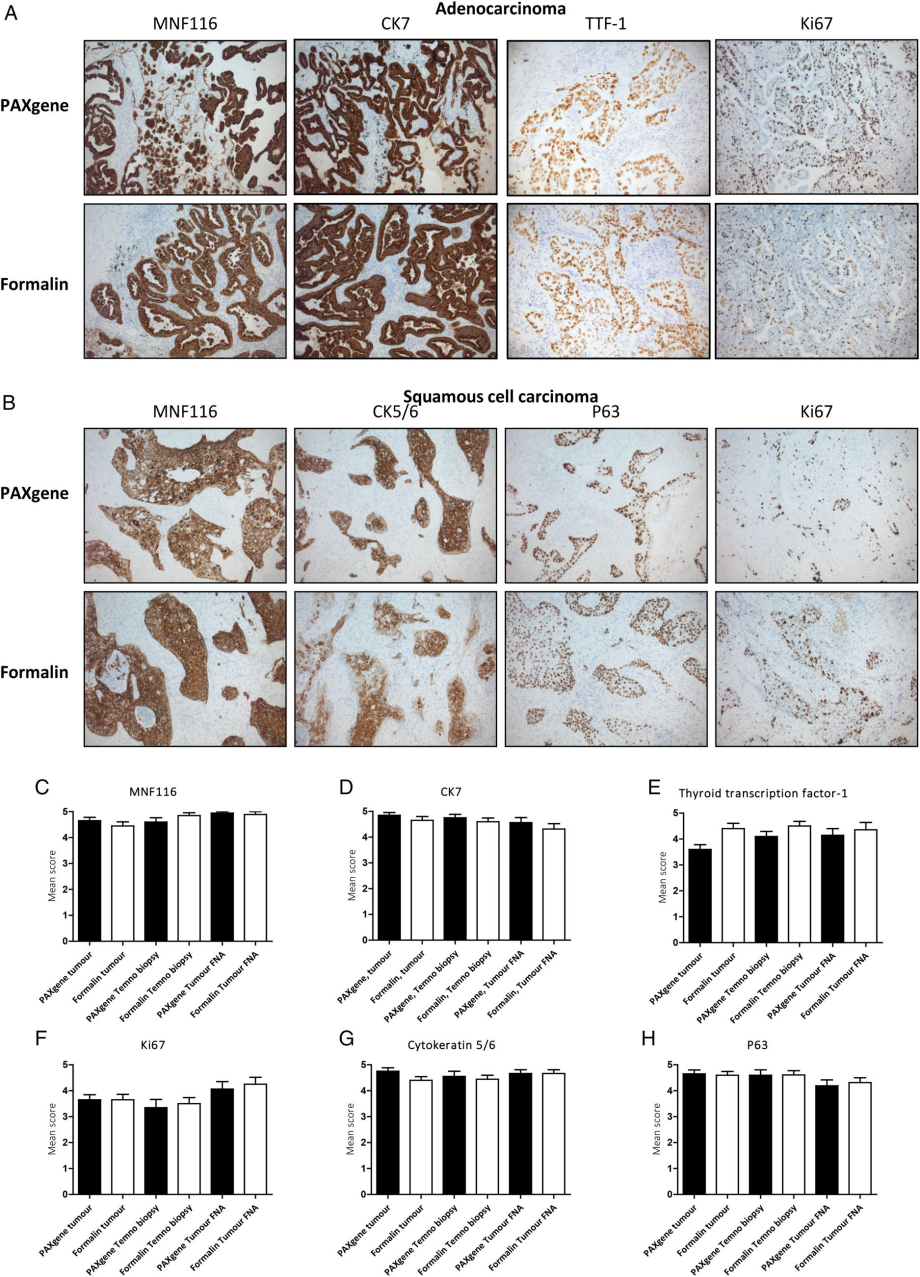
IHC staining. Representative images of PFPE and FFPE adenocarcinoma immunostained with MNF116, CK7, TTF‐1 and Ki67 (A) and squamous cell carcinoma immunostained with MNF116, CK5/6, p63 and Ki67 (B). IHC scores of MNF116 (C), CK7 (D), TTF‐1 (E), Ki67 (F), CK5/6 (G) and p63 (H) were comparable for PFPE and FFPE samples (*n* = 10).

H&E staining of PFPE and FFPE samples was of high quality and blind scores generally comparable (Figure 1A–F). Interestingly, blinded observers preferred nuclear H&E staining of PFPE biopsies and tumour FNA preparations. Representative histomicrographs of PFPE and FFPE adenocarcinoma or squamous cell carcinoma sections are shown in Figure 1G–J. Although no efforts were made to optimise immunohistochemical protocols for PFPE samples, and antigen retrieval was performed on all samples, immunohistochemistry was successful and mean scores were statistically similar. Representative immunostaining is demonstrated in Figure 2.

### QIAGEN analysis of DNA quality and integrity

**Figure 3:**
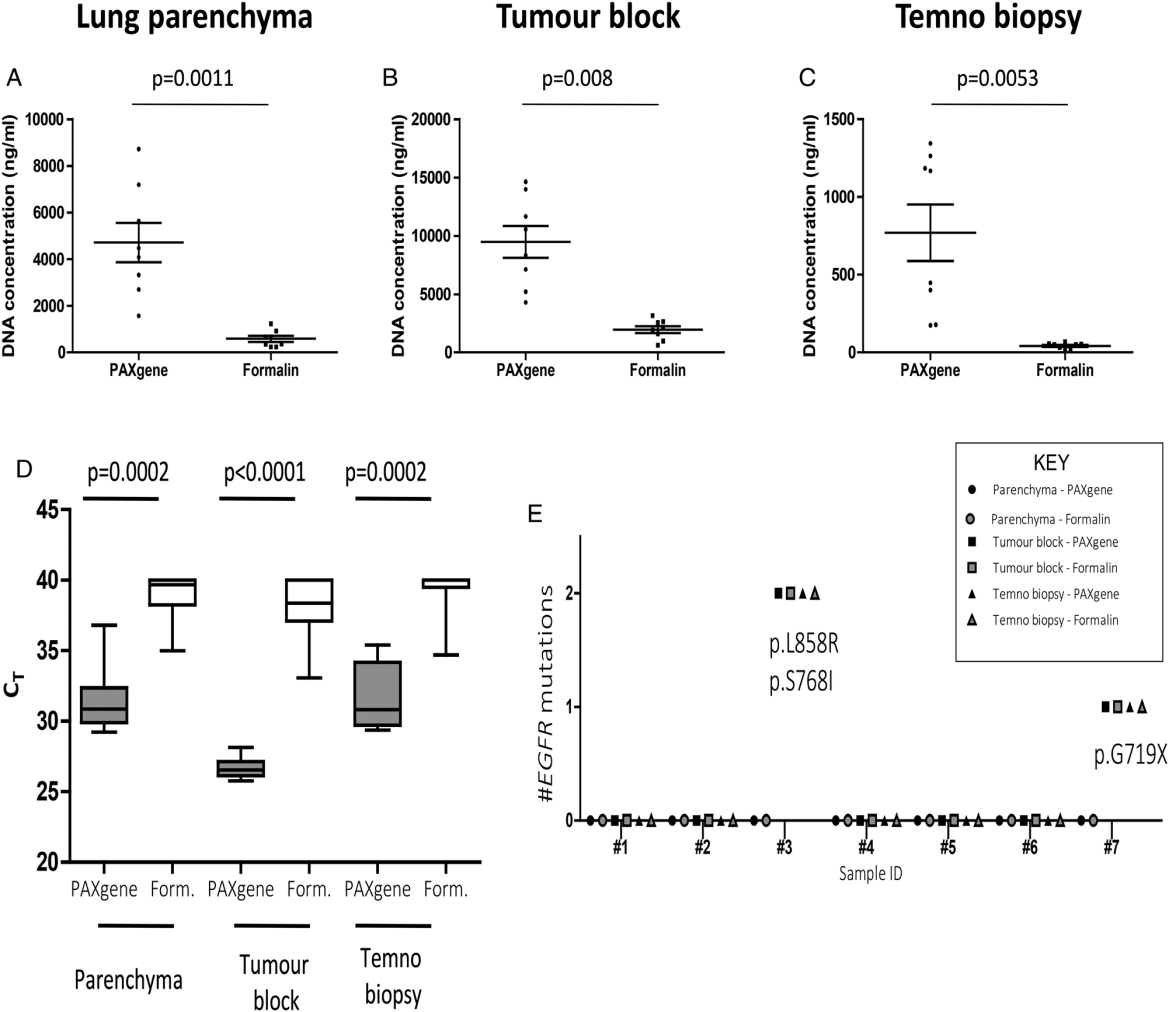
DNA analysis. DNA concentrations were greater in (A) PFPE lung parenchyma (4714 ± 839.3 ng versus 583.4 ± 125.9 ng, *p* = 0.0011), (B) PFPE tumour blocks, (9476 ng ±1371 ng versus 1955 ng ± 304.6 ng, *p* = 0.008) and (C) PFPE Temno biopsies (770.1 ± 181.7 ng versus 42.5 ± 7.44, *p* = 0.0053), than FFPE comparators. RNA RT PCR mean *C*_T_ values (*n* = 8) for β‐actin were lower (D) in PFPE lung parenchyma (31.48 ± 0.8484 versus 38.86 ± 0.6143, *p* = 0.002), block‐sized tumour samples (26.69 ± 0.2638 versus 38.01 ± 0.8508, *p* < 0.0001) and Temno biopsies (31.61 ± 0.8326 versus 39.24 ± 0.6544, *p* = 0.0002) compared to respective FFPE samples. *EGFR* mutation profiles were explored using the *EGFR* Therascreen® PCR assay (E). Paired samples from a single subject (Patient #8) were a technical failure and not included. Of the remaining *n* = 7 subjects, we identified a patient with a p. G719X *EGFR* mutation present in both PFPE and FFPE tumour blocks and Temno biopsies (and absent in both parenchyma blocks) and another subject harbouring two separate *EGFR* mutations (p.L858R and p.S768I) present in the block‐sized tumour samples and Temno biopsies but absent in both lung parenchyma indicating similar performance for PFPE and FFPE.

Mean DNA yields from all PFPE sample types were greater than FFPE comparators, with the larger block‐sized samples yielding more DNA for both parenchyma (Figure 3A, 4714 ± 839.3 ng versus 583.4 ± 125.9 ng, *p* = 0.0011) and for block‐sized pieces of lung tumour (Figure 3B, 9476 ± 1371 ng versus 1955 ± 304.6 ng, *p* = 0.008). Similarly, DNA yields were greater from PFPE Temno tumour biopsies compared to FFPE Temno biopsies (Figure 3C, 770.1 ± 181.7 ng versus 42.5 ± 7.44 ng, *p* = 0.0053). Mean cycle threshold *C*_T_ values for β‐actin RT‐PCR amplification were lower in PFPE lung parenchyma (31.48 ± 0.8484 versus 38.86 ± 0.6143, *p* = 0.002), block‐sized tumour samples (26.69 ± 0.2638 versus 38.01 ± 0.8508, *p* < 0.0001) and Temno tumour biopsies (31.61 ± 0.8326 versus 39.24 ± 0.6544, *p* = 0.0002) (Figure 3D).

### *therascreen*® *EGFR* PCR assay

Samples from a single subject (Patient #8) were a technical failure and not included. Of the remaining *n* = 7 subjects, we identified a patient with a p.G719X *EGFR* mutation present in both PFPE and FFPE tumour blocks and Temno tumour biopsies (and absent in the parenchyma block) and another subject harbouring two separate *EGFR* mutations (p.L858R and p.S768I) present in the block‐sized tumour samples and Temno biopsies and absent in the background lung parenchyma (Figure 3E).

### QIAGEN GeneReader™ NGS workflow and UMI

**Figure 4:**
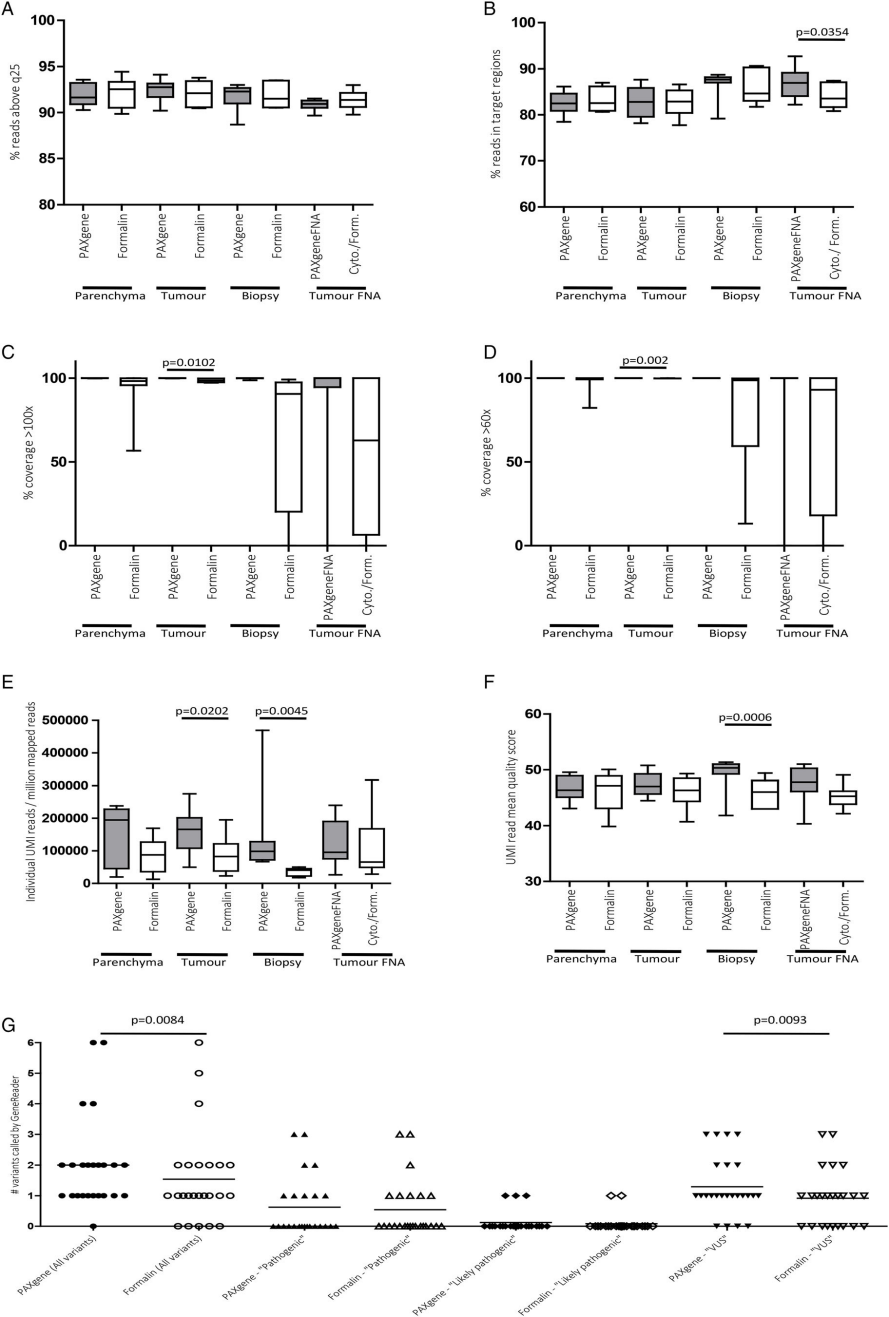
GeneReader™ NGS. Comparison of quality metrics for NGS indicated that mean quality scores for PFPE and FFPE samples were both good with over 90% of all samples having a *q* score > 20 (A) and all samples having a mean % of reads within target regions >80 and a significantly greater percentage of within‐target reads present in PFPE versus FFPE tumour FNA preparations (*p* = 0.0354) (B). Percentage coverage >100*x* (C) and >60*x* (D) was superior in PFPE samples and significantly so in Temno biopsies compared to FFPE counterparts (*p* = 0.0102 at >100*x* and *p* = 0.002 at >60*x* respectively). Greater numbers of individual UMI reads per million mapped reads were present in PFPE samples and reaching significance in PFPE tumour blocks (161 925 ± 24 464 versus 87 345 ± 19 776 individual reads/10^6^ mapped reads, *p* = 0.0105) and Temno biopsies (141 552 ± 47 553 versus 36 117 ± 4679 individual reads/10^6^ mapped reads, *p* = 0.0045 (E). Mean UMI read quality scores were higher in PFPE Temno biopsies compared to FFPE comparators (47.36 ± 0.7557 versus 46.03 ± 1.004, *p* = 0.0006) (F). The QIAGEN GeneReader™ analysis pipeline called a greater number of overall variants in PFPE samples than FFPE comparators (48 versus 37, *p* = 0.0084). Greater but non‐significant numbers of ‘pathogenic’ and ‘likely pathogenic’ variants were called in PFPE samples compared to FFPE samples (15 versus 13 and 3 versus 2 respectively). A significantly greater number of VUS were called in the PFPE samples compare to the FFPE equivalent (31 versus 22, *p* = 0.0093) (G).

We examined the suitability of paired samples for NGS by comparing common quality metrics (*q* scores, depth of coverage and read mapping efficiency) and used UMI technology to compare NGS performance of paired samples. Mean quality scores for PFPE and FFPE samples were generally good with over 90% of all reads having a *q* score > 25 (Figure 4A) and all samples having a mean % of reads within target regions >80 (Figure 4B). Percentage coverage >100*x* (Figure 4C) and >60*x* (Figure 4D) was superior in PFPE samples, reaching significance for PFPE Temno biopsies compared to FFPE counterparts (*p* = 0.0102 at >100*x* and *p* = 0.002 at >60*x* respectively). We compared the frequency of different UMI reads per million mapped UMI reads and found greater UMI read numbers in PFPE than FFPE comparators, reaching significance in PFPE tumour blocks and Temno biopsies (Figure 4E). In addition, the mean UMI read quality scores were higher in PFPE Temno biopsies compared to FFPE (Figure 4F).

### GeneReader™ NGS variant calling

We used the QIAGEN QCI analysis pipeline to call variants in PFPE and FFPE samples. A greater number of overall variants were detected in PFPE samples (*n* = 48) compared to the equivalent FFPE samples (*n* = 37, *p* = 0.0084). Of these total variants, there were *n* = 15 ‘pathogenic’ variants called in PFPE samples compared to *n* = 13 in the FFPE samples, *n* = 3 ‘likely pathogenic' variants in PFPE samples compared to *n* = 2 in FFPE samples, and *n* = 31 ‘variants of unknown significance’ (VUS) in PFPE samples compared to *n* = 22 VUS in the FFPE samples (*p* = 0.0093; Figure 4G).

### DNA extraction, quality metrics and targeted human lung cancer panel NGS at QUB

We prepared samples for DNA sequencing using the QIAseq targeted human lung cancer panel for NGS on Illumina platforms. Yields of extracted DNA were greater in PFPE lung parenchyma blocks (211.4 ± 40.72 ng/μl versus 123.5 ± 25.26 ng/μl, *p* = 0.042), tumour blocks (252.1 ± 44.0472 ng/μl versus 106.4 ± 16.1172 ng/μl, *p* = 0.0015), Temno biopsies (38.93 ± 4.648 ng/μl versus 13.68 ± 2.301 ng/μl, *p* = 0.0002) and PFPE tumour FNA preparations (20.8 ± 7.897 ng/μl versus 7.776 ± 2.972 ng/μl, *p* = 0.0431) measured by NanoDrop. Overall, values for DNA concentrations from QUBIT assessments were lower than those reported by NanoDrop but greater DNA concentrations were identified in PFPE Temno biopsies (17.08 ± 2.353 ng/μl versus 5.994 ± 1.438 ng/μl, *p* = 0.002) and PAXgene® FNA fixed tumour FNA samples (8.944 ± 2.358 ng/μl versus 2.621 ± 1.419 ng/μl, *p* = 0.005) than formalin or Cytolyt/formalin comparators. These findings are summarised in supplementary material, Figure S2.

**Figure 5:**
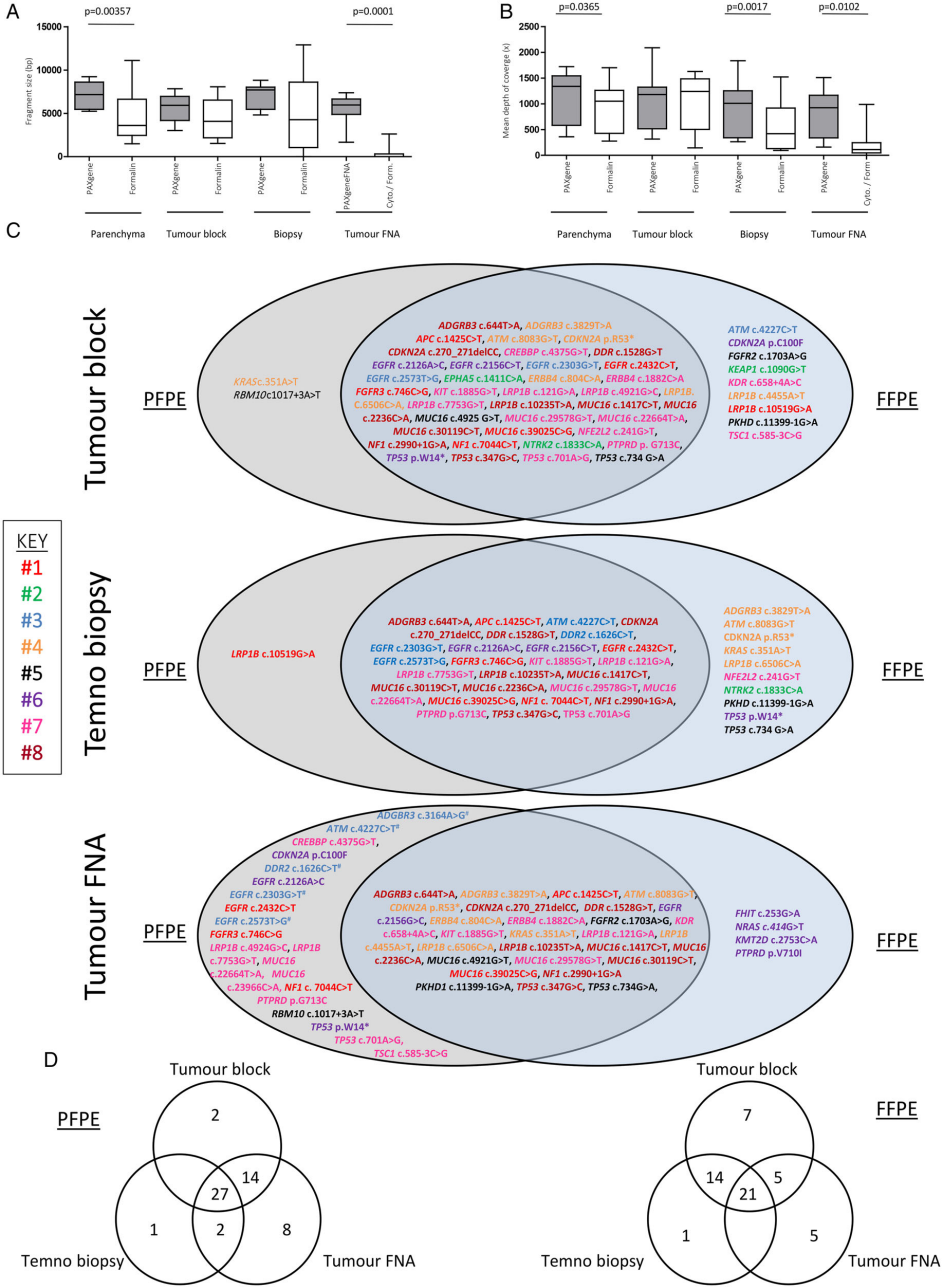
Illumina NGS. Mean fragment size was greater in PFPE lung parenchyma blocks (7069 ± 545.9 bp versus 4677 ± 1110 bp, *p* = 0.00357) and in tumour FNA preparations (5518 ± 626.2 bp versus 398.3 ± 320.0 bp, *p* = 0.0001) (A). Mean depth of coverage (*x*) was significantly deeper in PFPE parenchyma blocks (1165 ± 182.2*x* versus 951 ± 171.6*x*, *p* = 0.0365), Temno biopsies (1041 ± 194.4*x* versus 917.9 ± 196.7*x*, *p* = 0.0017) and in tumour FNA preparations (821.3 ± 166.7*x* versus 218.9 ± 114.0*x*, *p* = 0.0102) (B). All PFPE samples were suitable for NGS but *n* = 1 FFPE tumour FNA was deemed a technical failure. Venn diagrams summarising variant details and frequency in PFPE and FFPE tumour blocks, Temno biopsies and tumour FNA samples (C) and by sample type (D). We identified *n* = 4 *EGFR* mutations (*EGFR* c.2126A>C, *EGFR* c.2303G>T, *EGFR* c.2432C>T and *EGFR* c.2573T>G) in three individuals that were present in PFPE tumour FNA preparations but not identified in the FFPE comparator FNA. We should highlight that the FFPE tumour FNA from the subject harbouring *n* = 2 separate *EGFR* mutations was a technical failure and although we cannot exclude unintentional sampling variations, the potential underrepresentation of *EGFR* mutations in FFPE material is of note.

We found mean fragment size (Figure 5A) was greater in PFPE lung parenchyma blocks (7069 ± 545.9 bp versus 4677 ± 1110 bp, *p* = 0.00357) and in PFPE tumour FNA preparations (5518 ± 626.2 bp versus 398.3 ± 320.0 bp, *p* = 0.0001). We quantified mean depth of coverage (Figure 5B) and found that coverage (*x*) was significantly deeper in PFPE parenchyma blocks (1165 ± 182.2*x* versus 951 ± 171.6*x*, *p* = 0.0365), Temno biopsies (917.9 ± 196.7*x* versus 551.1 ± 182.8*x*, *p* = 0.0017) and tumour FNA preparations (821.3 ± 166.7*x* versus 218.9 ± 114.0*x*, *p* = 0.0102). All PFPE samples were suitable for NGS whilst *n* = 1 FFPE tumour FNA was deemed a technical failure. Concordance between PFPE and FFPE samples was generally good but genomic findings were not identical with genetic variants present in resections that were absent in the smaller biopsy and tumour FNA samples and vice versa. We have summarised these findings in Figure 5C and in supplementary material, Figure S3 and Table S4. A total of *n* = 41 genetic variants were identified in PFPE tumour blocks compared to *n* = 48 variants in FFPE tumour blocks. Of these, *n* = 39 genetic variants were common to both PFPE and FFPE tumour blocks, with *n* = 2 variants unique to the PFPE tumour block and *n* = 9 unique variants in the FFPE tumour block. For summary of variants detected by sample type (see Figure 5D). In regards to the detection of actionable *EGFR* mutations, both PFPE and FFPE tumour blocks performed equally with a total of *n* = 5 *EGFR* variants identified in both fixation types, present in 3 of 8 patients (37.5% of tumours sampled).

A total of *n* = 28 genetic variants were identified in PFPE Temno biopsies compared to *n* = 37 in the FFPE comparator Temno biopsies. In the PFPE Temno biopsies we detected an additional *n* = 3 variants absent in the corresponding PFPE tumour block, and *n* = 2 additional variants in the FFPE Temno biopsies that were absent in the corresponding FFPE tumour blocks. Only 60.96% of the variants present in the PFPE tumour block were also present in the corresponding PFPE Temno tumour biopsy and 72.92% of the variants identified in the FFPE tumour block were also present in the FFPE Temno biopsy. A single *LRP1B* variant was identified in the PFPE Temno biopsy that was absent in the FFPE Temno biopsy, compared to *n* = 10 variants present in the FFPE Temno biopsy but absent in the corresponding PFPE biopsy.

The PFPE tumour FNA samples contained *n* = 10 additional variants that were absent in the PFPE tumour blocks. Correspondingly, the FFPE tumour FNA samples contained *n* = 5 additional variants that were absent in the FFPE tumour blocks. In the PFPE tumour FNA preparations, a total of *n* = 48 genetic variants were identified compared to *n* = 32 in the FFPE tumour FNA samples. Critically, we identified *n* = 4 *EGFR* mutations (*EGFR* c.2126A>C, *EGFR* c.2303G>T, *EGFR* c.2432C>T and *EGFR* c.2573T>G) in three individuals that were present in PFPE tumour FNA preparations but not identified in the FFPE comparator FNA samples. We should highlight that the FFPE tumour FNA sample from the subject harbouring *n* = 2 separate *EGFR* mutations was a technical failure and although we cannot exclude unintentional sampling variations, the underrepresentation of *EGFR* mutations in FFPE material compared to those identified in PFPE material is noteworthy.

## Discussion

Molecular testing for driver genetic mutations to guide clinical decision making is a key strategy for improving survival for NSCLC patients 34 and demand for genomic studies of diagnostic samples is likely to increase. Despite these advances, reliance on formalin remains widespread and is highly problematic for genomic studies, as exemplified by the preference of fresh‐frozen tissue for the 100 000 genomes project. The reasons for the continued use of formaldehyde are to a degree historical and understandably cost‐related but, whilst formaldehyde‐based fixatives adequately preserve morphology, closer attention should be paid to over/under‐fixation of samples 21, 22, 23, 24, 25, and overcoming formalin or processing related artefacts for key molecular or genomic applications is increasingly challenging 35, 36, 37. At the time of publication, the cost of the PAXgene® fixative and stabiliser is <£14 per block and requires a separate formalin‐free tissue processor (or full change of reagents prior to each batched PAXgene® run). This may seem expensive compared to the cost of formalin but represents only a fraction of the cost of the investigation, diagnosis and treatment of a patient on a lung cancer care pathway. For context, a PET scan is in the order of £850 per scan 38 and Gefitinib >£2000/month (https://bnf.nice.org.uk/medicinal-forms/gefitinib.html). Moreover, DNA sequencing remains an expensive modality and improving the failure rate of poorly fixed or degraded samples brings significant benefits, including financial; and avoiding/reducing the need for repeat sampling and additional costs/burden of additional patient intervention is crucial.

In the study of lung cancer, techniques to maximise high quality DNA yield from small samples are particularly relevant; hence our inclusion of biopsy and tumour‐FNA cell blocks. We demonstrate that PAXgene® use is suitable for histopathological assessment and broadly comparable to formalin fixation for most common histology and immunohistochemical tests used for the study of lung cancer. Although PFPE H&E‐stained sections were more eosinophilic, we found no interpretive challenges with PFPE prepared samples, in line with previous studies of colon cancer 39, prostate histomorphology 40, and within the field of veterinary pathology 33. Immunostaining of PFPE sections was similar to FFPE stained sections (without tailoring optimisation or protocols), highlighting how immunostaining of PFPE samples could potentially be performed alongside FFPE samples with little laboratory impact. In addition to lung, immunohistochemistry of PFPE and FFPE samples has already been explored in a number of tissues including the prostate 40, and by tissue microarrays of lung and colon adenocarcinomas 41. Previously, a comprehensive immunohistochemical evaluation compared 28 different antibody clones raised against 14 different antigens (2 antibody clones per antigen). Somewhat in contrast to our findings, these studies found comparable staining in only 7 of 28 antibodies. A further 10 of 28 antibodies were found to be ‘interpretable but sub‐optimally immunostained’, with the remaining 11 of 28 antibodies (including TTF‐1) showing insufficient staining quality 41. We have made no efforts to optimise our PAXgene® immunohistochemistry and are confident that antibody sensitivity and specificity could be improved by antibody titration for PAXgene® samples or the omission of antigen retrieval (as this is not theoretically required as PAXgene® is non‐cross‐linking). In addition, previous studies to evaluate PAXgene® suitability for Immunohistochemistry and to optimise staining specifically for PAXgene®‐Tissue‐fixed samples have proved successful 41, 42, 43.

Our preliminary data demonstrates the feasibility of successful PD‐L1 immunostaining in paired PFPE and FFPE samples (see supplementary material, Figure S4). Whilst outside of the scope of this study, we acknowledge that suitability and equivalence of alternative fixatives for biomarker expression, including PD‐L1, is essential for lung cancer samples 44, 45, 46, 47. Although validation of PD‐L1 staining to date has been performed on FFPE material 45, further work exploring variations in PD‐L1 (and other emerging biomarkers, e.g. ROS1) expression and careful validation in samples fixed in reagents other than formalin is warranted.

In line with existing reports 33, 41, our quantification of DNA/RNA yield and quality indicates PFPE to be superior to FFPE samples. We also found that both DNA integrity and fragment size were superior in PFPE specimens, again in keeping with previous studies 41, 48. Our β‐actin RT‐PCR also supports these findings as we found that, on average, fewer PCR amplification cycles were required to exceed the background cycle threshold for PAXgene® Tissue fixed samples; supporting superior biomolecule preservation.

We panel‐sequenced extracted DNA using a combination of approaches including the QIAGEN GeneReader™ NGS workflow and Illumina sequencing. We utilised the use of UMI barcoding to evaluate the precise effects of formalin and PAXgene® fixation upon sequencing fidelity, a technology particularly useful for distinguishing true, low frequency emerging clones of tumour cells from sequencing or PCR artefacts. We identified greater numbers of high quality UMI‐reads in PFPE tumour blocks and in biopsies compared to FFPE samples. Whilst not a key objective of this study, we identified a large degree of heterogeneity between variants found in different sample types from individual patients (as well as between differently fixed paired samples). It is plausible that some differences could be attributed to intra‐tumour heterogeneity, and quicker or improved DNA fixation in smaller samples (relative to blocks) resulting in superior NGS performance.

Depth of coverage is a useful parameter to help understand how well DNA sequencing has performed and is particularly relevant to investigations of lung cancer as deeper sequencing increases confidence in calling low frequency variants. Mean depth of coverage was greater in PFPE samples compared with FFPE comparators; an effect more evident in the smaller sized PAXgene®‐fixed tumour‐FNA samples and likely facilitated by the presence of greater yields of high‐quality, non‐fragmented DNA in PFPE samples. Distinction between low‐frequency tumour clones and PCR or sequencing artefacts in lung cancer is crucial as the acquisition and detection of resistance mutations can dictate a switch to a second or third line agent 49. Critically, we identified a greater number of *EGFR* mutations in our PFPE tumour FNA samples (*n* = 4 mutations present in *n* = 3 patients) that were not detected in the FFPE comparator (although present in the FFPE tumour block and biopsy). Despite one of the FFPE tumour FNA specimens being a technical failure, this observation again supports increased variant calling sensitivity using PAXgene® fixation and we should consider that many clinical FNA samples are physically small, relatively poorly cellular and often need to be extensively tested. Whilst we accept that this is a relatively small study using surgical resection material to replicate sample types used for the diagnosis and staging of lung cancer, the possibility of missing *EGFR* mutations through reliance upon formalin fixation may have profound consequences for the clinical management of patients harbouring actionable findings. We also confirmed that actionable findings from NGS were more comprehensive than the RT‐PCR‐based *therascreen*® platform as the *EGFR* c.2432C>T (p.S811F) mutation in patient#1 and the *EGFR* c.2126G>C (p.E709A) mutation in patient #7 were not detected by *therascreen*® due to these primers not being included in the assay. In addition, the study of circulating cell free DNA (ccfDNA) for resistance mutations will also become critical in guiding patient management^,49,50^ and a number of approaches, including the use of PAXgene® blood collection tubes for ccfDNA stabilisation, have been evaluated 50, 51, 52, 53. Given its comprehensiveness, it is most likely that NGS will emerge as the preferential modality for studying genomic changes, regardless of original sample type or location and that appropriate fixation will be a critical factor for molecular analysis. Here we provide compelling evidence for improved variant calling (potentially including detection of actionable *EGFR* mutations in PFPE Tumour FNA preparations) by the use of PAXgene® fixation that were absent in FFPE comparators.

In summary, biomarker‐driven molecular stratification of patients with the detection of driver mutations and epigenetic alterations, longitudinal monitoring for the acquisition of resistance mutations and potential molecular screening for malignancy in at‐risk individuals (e.g. current smokers) are key approaches in the current and future management of NSCLC. NSCLC is a highly dynamic condition and a combination of treatment approaches, including chemotherapy, along with a biomarker‐ (e.g. PD‐L1 expression) or molecular‐guided first‐ or second‐line therapy followed by prospective testing of the tumour or ccfDNA to monitor acquisition of resistance mutations may be of benefit. Key to the clinical implementation of these strategies is optimal DNA stabilisation. Here we demonstrate that PAXgene® fixation is suitable for the majority of existing workflows (with little or no optimisation) and brings significant advantages for the molecular study of samples typical of those collected from lung cancer patients. Although we feel that PAXgene® specific workflows are not justified for the majority of samples, a duelled‐laboratory approach, whereby small samples including biopsies and FNA samples (i.e. those most likely to require molecular testing) are processed using formalin‐free protocols. We feel this approach will optimise DNA suitability for molecular analysis and will prove beneficial for many conditions including lung cancer, where patients often present with advanced disease and identification of actionable mutations is key to guide clinical management and improve patient survival.

## Author contributions statement

MS, TK, NC, PM, MS‐T, DG and DMR were involved in study design and conceived experiments. MS, TK, NC, MM, JG, PM, CM, EM, PS and SH carried out experiments and collected, analysed and interpreted data. MS and DR generated figures, searched literature and wrote the manuscript. All authors had final approval of the submitted and published versions.

## Disclaimers

GeneReader NGS System is intended for research use only. Not for use in diagnostic procedures. PAXgene® Tissue and FNA fixative are intended for research use only.

## Supporting information


**Supplementary materials and methods**
**Figure S1.** Summary of sample preparation, DNA extraction and sequencing and bioinformatic pipeline
**Figure S2.** DNA extraction and sample preparation for DNA sequencing using a QIAseq targeted human lung cancer panel
**Figure S3.** Summary of genetic findings from paired PFPE and FFPE tumour blocks, Temno tumour biopsies and tumour FNA samples
**Figure S4.** Representative PD‐L1 immunohistochemistry
**Table S1.** PAXgene® tissue processor schedule
**Table S2.** Formalin processing schedule
**Table S3.** Genes included on the human lung cancer QIAseq DNA panel
**Table S4.** Variant detection
