# Supplementary material for: Systematic evaluation of PAXgene® tissue fixation for the histopathological and molecular study of lung cancer
Source: J Pathol Clin Res. 2019 Nov 11;6(1):40–54. doi: 10.1002/cjp2.145 (PMC6966705; doi:10.1002/cjp2.145)
Supplement: Supplementary file 1 — Supplementary materials and methods Figure S1. Summary of sample preparation, DNA extraction and sequencing and bioinformatic pipeline Figure S2. DNA extraction and sample preparation for DNA sequencing using a QIAseq targeted human lung cancer panel Figure S3. Summary of genetic findings from paired PFPE and FFPE tumour blocks, Temno tumour biopsies and tumour FNA samples Figure S4. Representative PD‐L1 immunohistochemistry Table S1. PAXgene® tissue processor schedule Table S2. Formalin processing schedule Table S3. Genes included on the human lung cancer QIAseq DNA panel Table S4. Variant detection [file CJP2-6-40-s001.docx]

**Systematic evaluation of PAXgene tissue fixation for the histopathological and molecular study of lung cancer**

Southwood *et al J Pathol Clin Res* DOI 10.1002/cjp2.145

**Supplementary Materials and Methods**

**Plasma/Thrombin clot preparation**

FNA samples were centrifuged at 1500rpm/10mins, supernatant fixatives removed, and the pellets washed with distilled water for 15min. Samples were centrifuged again, supernatants removed and 0.5mL human plasma/0.5mL bovine thrombin added at room temperature, gently agitated until a plasma/thrombin clot formed, encapsulating the cellular pellet. Cytolyt-fixed clotted samples were placed into formalin (24-72hrs) and processed using a standard formalin-based schedule.

**Primers and probes:**

Forward 5´-TCA CCC ACA CTG TGC CCA TCT ACG A-3´; Reverse 5´-CAG CGG AAC CGC TCA TTG CCA ATG G-3´; Probe1 5´FAM-ATG CCC TCC CCC ATG CCA TCC TGC GT-3´BHQ; Probe 2 5´-Hex-ATG CCC TCC CCC ATG CCA TCC TGC GT-3´ BHQ QIAGEN.

**Supplementary Figures**

| **Day 1** | **Day 2** | **Day 3** | **Day 4** |
| --- | --- | --- | --- |
| DNA to be eluted in H_2_O | Fragmentation, end-repair and A-tailing  (45 min total incubation) | Universal PCR  (IL-Universal and IL-S502 primers) (1h 20 min total incubation) | Data Analysis – Download FASTQ file from BaseSpace |
| **↓** | **↓** | **↓** | **↓** |
| Fragment analyser assessment of FFPE gDNA | Adapter Ligation  (20 min total incubation) | 4^th^ QiaSeq Bead Clean Up  (40 min total incubation) | Data Analysis – Upload FASTQ file to Qiagen Data Analysis Centre |
| ↓ | ↓ | ↓ | ↓ |
| Qubit assessment of FFPE gDNA concentration | 1^st^ QiaSeq Bead Clean Up  (40 min total incubation) | QiaSeq Library Quant System  (120 min total incubation) | Download BAM and VCF files from Qiagen Data Analysis Centre |
| ↓ | ↓ | ↓ | ↓ |
| QuantiMIZE assessment of FFPE gDNA | 2^nd^ QiaSeq Bead Clean Up  (40 min total incubation) | Preparation of Pooling Datasheet  (20 min total incubation) | Qiagen IVA analysis of VCF files |
| ↓ | ↓ | ↓ | ↓ |
| QuantiMIZE assessment of DNA concentration of samples & +ve control) | Target Enrichment PCR  (Panel and IL-for primers)  (2 h total incubation) | Dilution and Pooling of Libraries  (20 min total incubation) | Mutation Report for Demultiplexed Samples |
| ↓ | ↓ | ↓ | ↓ |
| Preparation of fragmentation plate | 3^rd^ QiaSeq Bead Clean Up  (40 min total incubation) | Setting up MiSeq / NextSeq Run |  |

**Figure S1.** Summary of sample preparation, DNA extraction and sequencing and bioinformatic pipeline

p=0.0050

p=0.0002

**Lung parenchyma**

**Tumour block**

**Temno biopsy**

**Tumour FNA**

**A.**

**B.**

**C.**

**D.**

**E.**

**F.**

**G.**

**H.**

p=0.042

p=0.0015

p=0.0002

p=0.0431

**Figure S2.** DNA extraction and sample preparation for DNA sequencing using a QIAseq targeted human lung cancer panel. Concentrations of extracted DNA were greater in PFPE lung (A) parenchyma blocks (211.4±40.72ng/µl vs. 123.5±25.26ng/µl, p=0.042), (B) tumour blocks (252.1±44.0472ng/µl vs. 106.4±16.1172ng/µl, p=0.0015), (C) Temno biopsies (38.93±4.648ng/µl vs. 13.68±2.301ng/µl) and (D) tumour FNA preparations (20.8±7.897ng/µl vs. 7.776±2.972ng/µl) when measured by NanoDrop (Figure 5). QUBIT assessments of DNA concentrations were less remarkable although DNA concentrations were greater in PFPE (G) Temno biopsies (17.08±2.353ng/µl vs. 5.994±1.438ng/µl, p=0.002) and (H) Tumour FNA samples (8.944±2.358 ng/µl vs. 2.621±1.419 ng/µl, p=0.0102).

­­

PFPE

LRP1B c.1051G>A

FGFR3 c.746C>G,

APC c.1425C>T

EGFR c.2432C>T

NF1 c.7044C>T

MUC16 c.39025C>G

EGFR c.2303G>T, EGFR c2573T>G

EGFR c.2126A>C, EGFR c.2156G>C

*LRP1B* c.121G>A, *LRP1B* c.7753G>T, KIT c.1885G>T

*NFE2L2* c.241G>T, PTPRD p.G731C

TP53 c.701A>G, *MUC16* c.29578G>T

*MUC16* c.22664T>A, DDR2 c.1528G>T,

LRP1B c.10235T>A, ADGRB3 c.644T>A

*CDKN2A* c.270_271delCC

TP53 c.347G>C

NF1 c.2990+1G>A

MUC16 c.30119C>T

MUC16 c.2236C>A

MUC16 c.1417C>T

EPHA5 c.1411C>A

NTRK2 c.1833C>A

**Tumour**

**Tumour FNA**

**Temno Biopsy**

LRP1B c.6506C>A,

ERBB4 c.804c>A

ADRGRB3 c.3829T>A,

CDKN2A p.53*

ATM C8083G>T,

KRAS c.351A>T

TP53 c.734G>A,

MUC16 c.4925G>T

RBM10 c.1017+3A>T

TP53 p.W14*

*LRP1B* c.4921G>C

NFE2L2 c.241G>T

ERBB4 c.1882C>A

CREBP c.4375G>T

ADGRB3 c.3164A>G

LRP1B c.4455A>T

*PKHD* c.11399-1G>A

FGFR2 c.734G>A

CDKN2A p.C100F

MUC16 c.23966C>A

KDR c.658+4A>C

TSC1 c.585-3C>G

**Temno Biopsy**

**Tumour**

**Tumour FNA**

FFPE

LRP1B c.1051G>A, EPHA5 c.1411C>A,

KEAP1 c.1090G>T, CDKN2A p.C100F

***LRP1B* c.4921G>C,** CREBP c.4375G>T

TSC1 c.585-3C>G

FGFR3 c.746C>G,

EGFR c.2432C>T, NF1

c.7044C>T, NTRK2 c.1833C>A,

EGFR c.2303G>T,

EGFR c2573T>G,

ATM c.4227C>T,

EGFR c.2126A>C

TP53 p.W14*

***LRP1B* c.7753G>T**

***NFE2L2* c.241G>T**

**PTPRD p.G731C**

**TP53 c.701A>G**

***MUC16***

**c.22664T>A,**

APC c.1425C>T

MUC16 c.39025C>G

LRP1B c.6506C>A,

ADRGRB3 c.3829T>A

CDKN2A p.53*

ATM C8083G>T

*PKHD* c.11399-1G>A

TP53 c.734G>A,

EGFR c.2156G>C

***LRP1B* c.121G>A**

**KIT c.1885G>T**

***MUC16* c.29578G>T**

**DDR2 c.1528G>T**

**LRP1B c.10235T>A**

**ADGRB3 c.644T>A**

***CDKN2A* c.270_271delCC**

**TP53 c.347G>C**

**NF1 c.2990+1G>A**

**MUC16 c.30119C>T**

**MUC16 c.2236C>A**

**MUC16 c.1417C>T**

LRP1B c.4455A>T

ERBB4 c.804c>A

FGFR2 c.734G>A

MUC16 c.4925G>T

ERBB4 c.1882C>A

NRAS c.414G>T

FHIT C253G>A

PTPRD p.V710L

KMT2D c.2573C>A

KDR c.658+4A>C

KRAS c.351A>T

DDR2 c.1626C>T,

ATM c.4227C>T,

DDR2 c.1626C>T,

**Figure S3.** Summary of genetic findings from paired PFPE and FFPE tumour blocks, Temno tumour biopsies and tumour FNA samples.

**
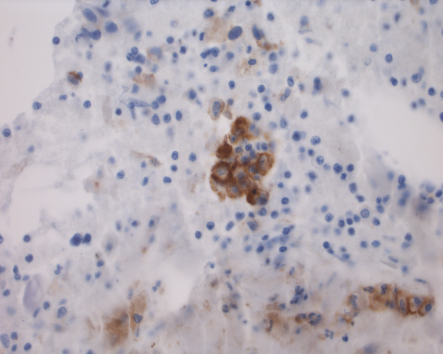

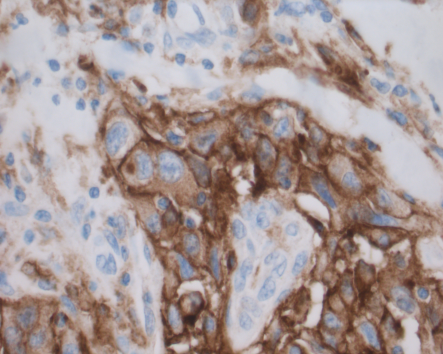

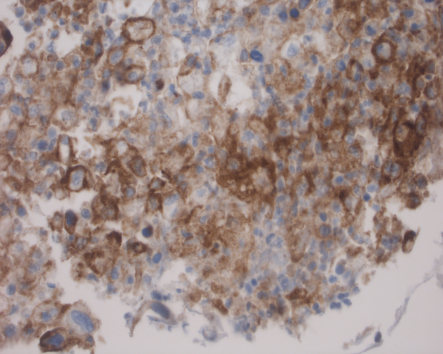

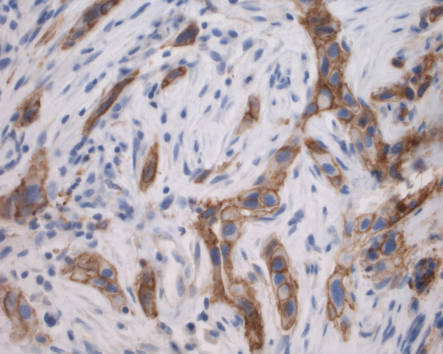
**

FFPE

PFPE

Tumour

block

Temno

biopsy

Tumour

FNA clot

A


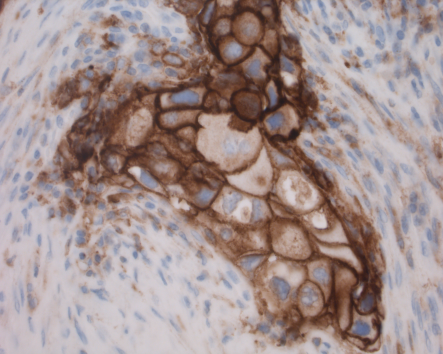


B


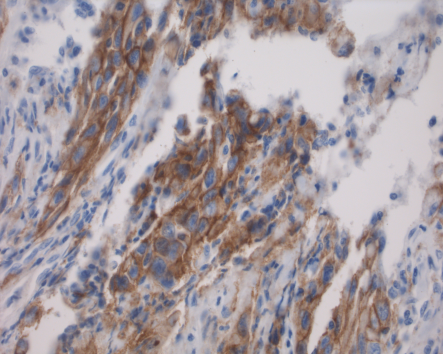


C

D

F

E

**Figure S4.** Representative PD-L1 immunohistochemistry in PFPE tumour blocks (A), Temno biopsies (C) and tumour FNA material (E), compared to paired, FFPE tumour blocks (B), Temno biopsies (D) and tumour FNA preparations (F). PDL-1 immunostaining was perfomed using a DAKO autostainer and DAKO PD-L1 22C3 pharmDx assay kit following the manufacturer’s protocol.

**Supplementary Tables**

**Table S1. PAXgene tissue processor schedule**

| **Position** | **Reagent** | **Time** | **Temperature** |
| --- | --- | --- | --- |
| 1 | N/A | N/A | N/A |
| 2 | 80% IMS | 4-52 hours | 15-25°C |
| 3 | 90% IMS | 60mins | 15-25°C |
| 4 | 99% IMS | 60mins | 15-25°C |
| 5 | 99% IMS | 60 mins | 15-25°C |
| 6 | 99% IMS | 60 mins | 15-25°C |
| 7 | 50%/50% IMS / Xylene | 60 mins | 15-25°C |
| 8 | Xylene | 60 mins | 15-25°C |
| 9 | Xylene | 60 mins | 15-25°C |
| 10 | Xylene | 60 mins | 15-25°C |
| 11 | Paraffin wax | 60 mins | 60°C |
| 12 | Paraffin wax | 60 mins | 60°C |

**Table S2. Formalin processing schedule**

| **Position** | **Reagent** | **Time** | **Temperature** |
| --- | --- | --- | --- |
| 1 | Neutral Buffered Formalin | 4-52 hours | 15-25°C |
| 2 | 80% IMS | 60mins | 15-25°C |
| 3 | 90% IMS | 60mins | 15-25°C |
| 4 | 99% IMS | 60mins | 15-25°C |
| 5 | 99% IMS | 60 mins | 15-25°C |
| 6 | 99% IMS | 60 mins | 15-25°C |
| 7 | 50%/50% IMS / Xylene | 60 mins | 15-25°C |
| 8 | Xylene | 60 mins | 15-25°C |
| 9 | Xylene | 60 mins | 15-25°C |
| 10 | Xylene | 60 mins | 15-25°C |
| 11 | Paraffin wax | 60 mins | 60°C |
| 12 | Paraffin wax | 60 mins | 60°C |

**Table S3. Genes included on the human lung cancer QIAseq DNA panel**

| *AKT1* | *ALK* | *AMER1* | *APC* | *ARID1A* | *ATM* | *BA13* | *BAP1* | *BRAF* | *CDKN2A* |
| --- | --- | --- | --- | --- | --- | --- | --- | --- | --- |
| *CDKN2B* | *CREBBP* | *CTNNB1* | *DDR2* | *EGFR* | *EPHA5* | *ERBB2* | *ERBB4* | *FBX07* | *FBXW7* |
| *FGFR1* | *FGFGR2* | *FGFR3* | *FHIT* | *GRM8* | *HRAS* | *JAK2* | *KDR* | *KEAP1* | *KIT* |
| *KMT2D* | *KRAS* | *LRP1B* | *MAP2K1* | *MDM2* | *MET* | *MGA* | *MLH1* | *MUC16* | *MYC* |
| *NFE2L2* | *NF1* | *NOTCH1* | *NRAS* | *NTRK1* | *NTRK2* | *NTRK3* | *PDGFRA* | *PIK3CA* | *PIK3CG* |
| *PIK3R1* | *PIK3R2* | *PKHD1* | *PTEN* | *PTPRD* | *RARB* | *RASSF1* | *RB1* | *RBM10* | *RET* |
| *RIT1* | *ROS1* | *RUNX1T1* | *SETD2* | *SMAD4* | *SMARCA4* | *SOX2* | *STK11* | *TNFAIP3* | *TP53* |
| *TSC1* | *U2AF1* |  |  |  |  |  |  |  |  |

**Table S4. Variant detection**

|  | | **PAXgene, n (%)** | **Formalin, n (%)** |
| --- | --- | --- | --- |
| **Tumour block** | Variants detected | 41 | 48 |
| **Temno biopsy** | Variants detected | 28 | 37 |
|  | Unique variants (not present in tumour block) | 3 (10.71% of those in Temno biopsy) | 2 (5.40% of those in Temno biopsy) |
|  | Variants in Temno biopsy also present in tumour block | 25 (60.96% of those in tumour block) | 35 (72.92% of those in tumour block) |
| **Tumour FNA** | Variants detected | 48 | 32 |
|  | Unique variants (not present in tumour block) | 10 (20.83% of those present in tumour FNA) | 5 (15.62% of those present in tumour FNA) |
|  | Variants in Tumour FNA also present in tumour block | 39 (95.12% of those in tumour block) | 27 (56.25% of those in tumour block) |
